# Self-replication of a quantum artificial organism driven by single-photon pulses

**DOI:** 10.1038/s41598-021-96048-6

**Published:** 2021-08-12

**Authors:** Daniel Valente

**Affiliations:** Instituto de Física, Universidade Federal de Mato Grosso, Cuiabá, Mato Grosso 78060-900 Brazil

**Keywords:** Quantum information, Single photons and quantum effects, Theoretical physics, Quantum physics, Statistical physics, Thermodynamics

## Abstract

Imitating the transition from inanimate to living matter is a longstanding challenge. Artificial life has achieved computer programs that self-replicate, mutate, compete and evolve, but lacks self-organized hardwares akin to the self-assembly of the first living cells. Nonequilibrium thermodynamics has achieved lifelike self-organization in diverse physical systems, but has not yet met the open-ended evolution of living organisms. Here, I look for the emergence of an artificial-life code in a nonequilibrium physical system undergoing self-organization. I devise a toy model where the onset of self-replication of a quantum artificial organism (a chain of lambda systems) is owing to single-photon pulses added to a zero-temperature environment. I find that spontaneous mutations during self-replication are unavoidable in this model, due to rare but finite absorption of off-resonant photons. I also show that the replication probability is proportional to the absorbed work from the photon, thereby fulfilling a dissipative adaptation (a thermodynamic mechanism underlying lifelike self-organization). These results hint at self-replication as the scenario where dissipative adaptation (pointing towards convergence) coexists with open-ended evolution (pointing towards divergence).

## Introduction

The longstanding question concerning the general principles of life still inspires a large diversity of concepts, methods and viewpoints^1–4^. Besides its fundamental value, for instance towards a universal biology^5^, looking for generalities could eventually enable us to imitate and extend the sophisticated dynamics achieved by living things. The artificial life approach, for instance, circumvents the biochemical constraints of living organisms by studying computer programs that mimic distinctive features of life. Artificial organisms can harness self-replication so as to mutate, compete and evolve complex features^6,7^. When one is interested in the most essential aspects of artificial life, quantum mechanics can be instrumental in casting the computation as operations on a handful of quantum bits^8–11^. Still, the finely engineered character of the computers assumed from the outset leaves open the question of what principles could explain the self-assembly of lifelike information-processing systems from physical laws.

The nonequilibrium thermodynamic approach, by contrast, looks for the emergence of lifelike behaviours in driven physical systems, comprising disordered, self-assembling, and self-replicating ones^4,12–20^. Remarkably, it has been recently proposed that nonequilibrium thermodynamics could generalize Darwinian evolution, even for non-replicating systems^21^. The idea is that exceptional specialization, or fine-tuned adaptation, to an environment by a fluctuating physical system can be fueled by the irreversible work consumption along far-from-equilibrium trajectories^22,23^. This exceptional self-organized response of a driven system to the patterns of its environment has been called dissipative adaptation^4,13,20^. Quantum physics can, once more, help us to showcase the most elementary aspects of dissipative adaptation^24^. Yet, thermodynamic studies of adaptation have been focusing mostly on systems that stabilize in the out-of-equilibrium states, lacking a more thorough discussion on the origins of diversification^25^ and open-ended evolution^7,26^ akin to that achieved with artificial life, and necessary for explaining the ever-growing complexity and diversity of biological species.

Can a driven physical system undergoing dissipative adaptation implement an artificial-life computational operation? This would provide a further step in the connections between the information-processing underlying self-replication and the dissipation of energy inherent to metabolism^27–29^, ultimately helping us to imitate the transition from inanimate to living matter^2,30^ in diverse physical systems. More broadly, searching for a lifelike self-organized computation could also contribute to the recently proposed goal of ‘thermodynamic computing’^31^.

Here, I formulate a toy model inspired by this idea of an artificial-life code with a driven system undergoing dissipative adaptation. Namely, the self-replication of a minimal quantum artificial organism starts due to single-photon pulses added to a zero-temperature environment. The model results in a replication probability that is linearly proportional to the average work absorbed by the replicating organism, characterizing a quantum dissipative adaptation. Counterintuitively, spontaneous mutations cannot be completely suppressed even in the zero-temperature limit, since they are induced by the driving pulses themselves. Mutations are rare, as they happen far from resonance. Because mutations may lead to open-endedness, the model thus hints at a possible connection between dissipative adaptation and open-ended evolution that has not been discussed so far, to the best of my knowledge.

The significance of this paper is mainly to bridge some aspect of artificial life and nonequilibrium thermodynamics. The quantum mechanical framework has been chosen so as to keep things in terms of basic resources such as atoms, photons, and their interactions. It turns out that the model may be thought of as illustrative of Walker and Davies viewpoint^30^, in that genetics is a digital form of information processing (simulated here with quantum bits), to be contrasted with the analogue form of information processing in metabolism (simulated here with continuous pulses). From a quantum information perspective, this paper can be seen as the quantum thermodynamics of a quantum cloning process^32–35^ driven by single photons. Even though the words ‘cloning’ and ‘self-replication’ can be used interchangeably here, the option for the latter is motivated by the original goal of mimicking lifelike information processing with an out-of-equilibrium physical system.

## Results

### Quantum artificial organism

The quantum organism is defined as a chain of quantum systems, as inspired by the polymeric structure of nucleic acid strands (DNA or RNA). Each quantum system has three energy levels. We choose a lambda configuration, so as to guarantee that the two lowest-energy levels are stable in the zero-temperature limit. The two lowest-energy levels are labeled here as $|a_n\rangle _1$ and $|b_n\rangle _1$, where *n* refers to the *n*-th lambda system in the chain, and the index 1, to the original gene (the original chain). States $|a_n\rangle _1$ and $|b_n\rangle _1$ play the role of two possible equivalents of nucleotide bases (instead of four, as in DNA or RNA). The original quantum artificial gene is defined by a (generally aperiodic) sequence of these lowest-energy states (a string), let us say

1 $$\begin{aligned} |\mathrm {gene}\rangle _1 = |a_1\rangle _1|b_2\rangle _1 \ ... \ |a_N\rangle _1. \end{aligned}$$

*N* here is the gene size, also corresponding to the chain size. Quantum superpositions of bases in $|\mathrm {gene}\rangle _1$ are not considered in this paper, as further explained below.

### Self-replication

The idea is to look for a dynamical process *U* (a global organism-plus-environment unitary dynamics), so that

2 $$\begin{aligned} U|\mathrm {gene}\rangle _1 |\mathrm {bases}\rangle _2 |\mathrm {source}\rangle \rightarrow |\mathrm {gene}\rangle _1 |\mathrm {gene}\rangle _2 |\mathrm {source}'\rangle , \end{aligned}$$

where *U* must be independent of the initial state $|\mathrm {gene}\rangle _1$. In (2), $|\mathrm {bases}\rangle _2 = \prod _{n=1}^N |b_n\rangle _2$ is the state representing the available environment bases upon which the organism can act to compose the copied gene, $|\mathrm {gene}\rangle _2$. States $|b_n\rangle _2$ here are the fundamental states of the lambda systems (therefore, their thermal equilibrium states in the limit of zero temperature). The source state

3 $$\begin{aligned} |\mathrm {source}\rangle = \prod _{n=1}^N |s_n\rangle \end{aligned}$$

describes the initial state of the environment degrees of freedom that provide the energy source; by the end of the process, the environment may have been modified to some final state $|\mathrm {source}'\rangle = \prod _n |s_n'\rangle$. Importantly, the nucleic-acid analogy guides us to look for a process $U =U(\left\{ r_n \right\} )$, which depends on the free parameters $\left\{ r_n \right\}$ symbolizing spatial distances between each gene base (the template lambda system) and its corresponding environment base (the environment lambda system which undergoes the copying process). In other words, the ability of the template lambda system to copy itself shall depend on the distance to the environment base.

**Figure 1:**
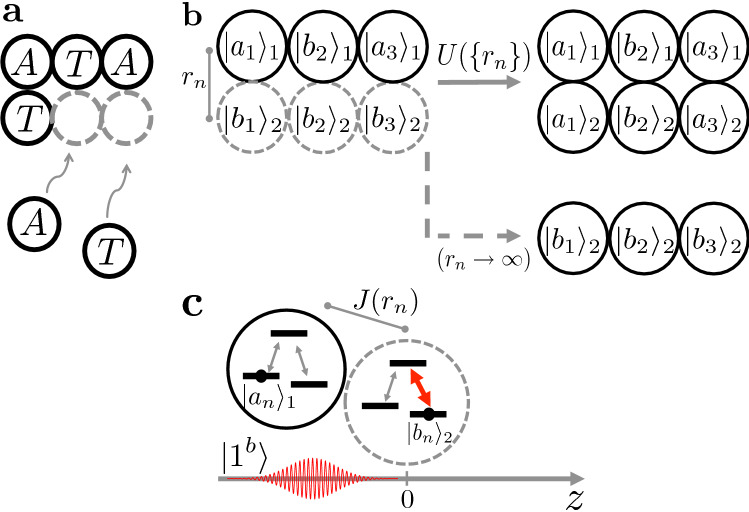
Natural versus artificial gene self-replication. (**a**) Simplified picture of a self-replicating biological gene. A single nucleic acid strand forms the original gene with nucleotide bases *A*, *T* and *A* . The complementary environment bases, *T*, *A* and *T*, form the copy. In the process, the bare states (dashed gray circles) undergo a transformation to the filled states (full black circles) dictated by the original gene (curved arrows). (**b**) A sequence of states $|a_1\rangle _1|b_2\rangle _1|a_3\rangle _1$ (full black circles) specifies the original quantum artificial gene. The environment bases are initially set to their ground states, $|b_n\rangle _2$, for $n = 1,2,3$ (dashed gray circles), which can be regarded as bare states. In the process, the bare states undergo a transformation to a filled state (full arrow), dictated by (and ideally identical to) the original gene state, $|a_1\rangle _1|b_2\rangle _1|a_3\rangle _1 \otimes |b_1\rangle _2|b_2\rangle _2|b_3\rangle _2 \rightarrow |a_1\rangle _1|b_2\rangle _1|a_3\rangle _1 \otimes |a_1\rangle _2|b_2\rangle _2|a_3\rangle _2$. This transformation is described by a unitary operator $U(\left\{ r_n \right\} )$ for the global organism-plus-environment dynamics, including that of the source of energy. The unitary operator $U(\left\{ r_n \right\} )$ depends on the free parameter $r_n$, representing the distance between the *n*-th gene base and its corresponding environment base. If $r_n \rightarrow \infty$, the environment bases are expected to be left unchanged (dashed arrow). (**c**) Inspired by the idea that sunlight has been crucial to the transition from inanimate to living matter, here each single-photon pulse (red wavepacket described by the state $|1^b\rangle$) drives (activates) a single environment base (lambda system in dashed gray circle). The pulse propagates towards the positive *z* direction and couples states $|b_n\rangle _2$ and $|e_n\rangle _2$ (red thicker double arrow). This light-matter dynamics depends on the couplings $J(r_n)$ between each original base (lambda system in full black circle) and the corresponding environment base.

From now on, replication is assumed to be modular, that is, the replication of each gene unit ($| \bullet _n \rangle _1$) is independent of the other units, $U(\left\{ r_n \right\} ) = \prod _n U_n(r_n)$, where $[U_n,U_m] = 0$ for all *m*, *n*. This assumption is motivated by the modular character of the self-replication of nucleic acids. A perfect replication transition should now read

4 $$\begin{aligned} U_n |a_n\rangle _1|b_n\rangle _2|s_n\rangle \rightarrow |a_n\rangle _1 |a_n\rangle _2 |s_n'\rangle , \end{aligned}$$

and a perfect dormant transition,

5 $$\begin{aligned} U_n |b_n\rangle _1|b_n\rangle _2|s_n\rangle \rightarrow |b_n\rangle _1 |b_n\rangle _2 |s_n\rangle , \end{aligned}$$

for $n = 1, ... , N$. To guarantee that a gene base does not affect an infinitely far apart environment base, we look for a unitary that satisfies $U_n(r_n\rightarrow \infty ) \rightarrow 1$. See Fig. 1.

Arbitrary superpositions of $|a_n\rangle _1$ and $|b_n\rangle _1$ cannot be copied with an arbitrarily high fidelity (quality), as states the so called no-cloning theorem^32–35^. The theorem assumes, however, that it is perfectly possible in principle to do so for a particular (preferred) orthonormal basis. This explains our choice in Eq. (1). Here, we intend to find the best replicator allowed by nature (by quantum mechanics, in this case), with the goal of verifying whether it does consume a maximal amount of work. In other words, we look for a model for $U_n$, trying to fulfill the perfect cloning as allowed in principle for the eigenenergies of the lambda systems.

### Organism-plus-environment Hamiltonian

We assume an autonomous dynamics

6 $$\begin{aligned} U_n = \exp {\left( -i H t /\hbar \right) }, \end{aligned}$$

where the time-independent global Hamiltonian *H*, describing the organism plus the environment, is given by

7 $$\begin{aligned} H = H_S + H_I + H_E. \end{aligned}$$

*H* does not depend on the index *n*, meaning that the dynamics of each base follows the same rules. The system Hamiltonian, $H_S$, describes the original gene base only. $H_E$ describes a composite environment, $H_E = H_B + H_F$, where $H_B$ is the environment base Hamiltonian, and $H_F$ is the electromagnetic field Hamiltonian. Correspondingly, the interaction Hamiltonian $H_I$ is also composite, $H_I = H_{SB} + H_{BF}$, where $H_{SB}$ describes the interaction of the original gene base with the environment base, and $H_{BF}$ describes the interaction of the environment base with the electromagnetic field.

The explicit expressions for the Hamiltonians are as follows. First,

8 $$\begin{aligned} H_S = \hbar \omega _b^{(0)} |e_n\rangle _1 \langle e_n|_1 + \hbar \delta _a^{(0)} |a_n\rangle _1 \langle a_n|_1, \end{aligned}$$

where $|e_n\rangle _1$ is the excited state of the *n*-th lambda system in the original artificial organism. Analogously, the environment base Hamiltonian reads

9 $$\begin{aligned} H_B = \hbar \omega _b^{(0)} |e_n\rangle _2 \langle e_n|_2 + \hbar \delta _a^{(0)} |a_n\rangle _2 \langle a_n|_2, \end{aligned}$$

showing that all lambda systems have been chosen identical to one another.

The coupling Hamiltonian between the system (the original gene base) and the environment base is

10 $$\begin{aligned} H_{SB} = -J \ \sigma _{a1}^{(z)} \sigma _{a2}^{(z)}, \end{aligned}$$

where $\sigma _{ai}^{(z)} = |e_n\rangle _i\langle e_n|_i - |a_n\rangle _i\langle a_n|_i$. $H_{SB}$ allows the gene base state to act as a switch to the dynamics of the environment base. As will become clearer below, the gene can selectively induce the environment base to become energetically resonant with the electromagnetic field, reminding us of an enzyme-like effect. For the degree of influence to depend on the distance parameters, we consider $J = J(r_n)$. Condition $U_n(r_n\rightarrow \infty ) = 1$ suggests that $J(r_n\rightarrow \infty ) = 0$. Ising-type couplings such as the one used here find widespread applications in protein physics^36,37^. Also, similar position-dependent dipole-dipole couplings generalizing the van der Waals forces have been proposed as a means for constructing biologically-inspired quantum molecular machines, which actively and autonomously self-protect their quantum data against noise from pre-existing non-engineered environments^38,39^.

The base-field interaction Hamiltonian is given by a dipolar coupling, in the rotating-wave approximation^24,35,40^,

11 $$\begin{aligned} H_{BF} = -i\hbar g \sum _\omega (a_\omega \sigma _{a2}^\dagger + b_{\omega } \sigma _{b2}^\dagger - \text{ H.c.}). \end{aligned}$$

The continuum of frequencies, $\sum _\omega \rightarrow \int d\omega \varrho _\omega \approx \varrho \int d\omega$, gives rise to the dissipation rate $\Gamma = 2\pi g^2 \varrho$, in the Wigner-Weisskopf approximation. Modes $\left\{ a_\omega \right\}$ and $\left\{ b_\omega \right\}$ represent orthogonal quantized field modes. The raising operators read $\sigma _{a2}^\dagger = |e_n\rangle _2 \langle a_n|_2$ and $\sigma _{b2}^\dagger = |e_n\rangle _2 \langle b_n|_2$. $\text{ H.c. }$ is the Hermitian conjugate. The choice for coupling the field only to the environment base ($|\bullet _n \rangle _2$) is justified by the fact that all lambda systems are identical in this model, hence it should ideally make no difference what base the photon hits each time. The rotating-wave approximation is useful here so as to keep the calculations restricted to the one-excitation subspace. Finding the consequences of more general light-matter couplings on self-replication, in the spirit of recent light-harvesting studies^41,42^, seems an interesting direction for further investigations. Finally, the field Hamiltonian is $H_F = \sum _\omega \hbar \omega (a_\omega ^\dagger a_\omega + b_\omega ^\dagger b_\omega )$, also considered in the continuum of frequencies limit.

### Single-photon pulses as the energy source

The source of energy for the self-replication is the initial out-of-equilibrium state of the electromagnetic environment. This is inspired by the reasonable hypothesis that sunlight may have played a significant role in the transition from inanimate to living matter. To make it the most elementary energy source, we consider in Eq. (3) single-photon pulses added to a zero-temperature background^24^, so that

12 $$\begin{aligned} |s_n\rangle = |1^b\rangle \equiv \sum _\omega \phi _\omega ^b(0) b_\omega ^\dagger |0\rangle . \end{aligned}$$

$|0\rangle = \prod _\omega |0_\omega ^a\rangle |0_\omega ^b\rangle$ is the vacuum state of all the field modes. Modes $\left\{ a_\omega \right\}$ are not initially populated, so as to maximize the irreversibility of the self-replication. The single-photon pulse admits a one-dimensional real-space representation (see Fig. 1c.),

13 $$\begin{aligned} \phi (z,t) \equiv \sum _\omega \phi _\omega ^b(t) \exp (i k_\omega z), \end{aligned}$$

where $k_{\omega } = \omega /c$, and *c* is the speed of light. As before, the sum over modes is to be considered in the continuum of frequencies limit. The pulse can also be decomposed as

14 $$\begin{aligned} \phi (z,t) = \phi ^{\mathrm {e}}(z,t) \exp {[-i\omega _L (t - z/c)]}, \end{aligned}$$

in terms of its central frequency $\omega _L$ and its envelope function $\phi ^{\mathrm {e}}(z,t)$.

To keep the spirit of an autonomous scenario, the photon pulse is assumed to have been spontaneously emitted from an arbitrarily distant source (not considered in the Hamiltonian). This implies an exponential shaped envelope

15 $$\begin{aligned} \phi ^{\mathrm {e}}(z,0) = \mathcal {N} \Theta (-z) \exp {\left[ \Delta z / (2c) \right] }. \end{aligned}$$

$\Delta$ here is the pulse spectral linewidth. $\mathcal {N} \equiv \sqrt{2\pi \varrho \Delta }$, a normalization constant. $\Theta (z)$, the Heaviside step function. The transition frequency of this hypothetical distant emitter corresponds to $\omega _L$, in Eq. (14), and its natural linewidth, to $\Delta$. Most importantly, $\omega _L$ shall be regarded here as a fixed constant, rather than a free-varying parameter. This is analogous to the idea of a steady peak in the sunlight spectrum. By contrast, the spectral linewidth $\Delta$ of each photon pulse may vary, so as to mimic a kind of disorder in the natural linewidths in the hypothetical ensemble of distant single-photon emitters.

### Global organism-plus-environment dynamics

The global dynamics can be described by the state

16 $$\begin{aligned} |G_k(t)\rangle = \exp (-iHt/\hbar ) |G_k(0)\rangle , \end{aligned}$$

where *H* is given by Eq. (7), and $|G_k(0)\rangle = |k_n\rangle _1|b_n\rangle _2|1^b\rangle$, for $k=a,b$.

Due to conservation in the number of excitations, the time-dependent state can be written for $k=a$ as

17 $$\begin{aligned} |G_a(t)\rangle = \sum _\omega F_\omega (t) |a_n\rangle _1|b_n\rangle _2|1_\omega ^b\rangle + R_e(t) |a_n\rangle _1|e_n\rangle _2|0\rangle + \sum _\omega R_{a \omega }(t) |a_n\rangle _1|a_n\rangle _2|1_\omega ^a\rangle . \end{aligned}$$

$F_{\omega }$ describes a failed replication, leaving a photon at mode $b_{\omega }$. $R_{e}$ describes a replication transient excitation, leaving the field in the vacuum state. $R_{a \omega }$ describes a replication accomplishment, leaving a photon at mode $a_{\omega }$.

Accordingly, the global state can be written for $k=b$ as

18 $$\begin{aligned} |G_b(t)\rangle = \sum _\omega D_\omega (t) |b_n\rangle _1|b_n\rangle _2|1_\omega ^b\rangle + M_e(t) |b_n\rangle _1|e_n\rangle _2|0\rangle + \sum _\omega M_{a \omega }(t) |b_n\rangle _1|a_n\rangle _2|1_\omega ^a\rangle . \end{aligned}$$

$D_\omega$ describes a dormant state, leaving a photon at mode $b_\omega$. $M_e$ describes a mutation transient excitation, leaving the field in the vacuum state. $M_{a \omega }$ describes an (undesirable) mutation accomplishment, leaving a photon at mode $a_\omega$. The initial state of the field implies that $F_\omega (0) = \phi ^{b}_\omega (0)$ or $D_\omega (0) = \phi ^{b}_\omega (0)$, as defined in Eq. (12), depending on the initial state of the original gene base. The equations of motion are shown in the Methods.

### Transition probabilities

The organism replication probability is defined here as $p_{a_1,b_2 \rightarrow a_1,a_2}(t) \equiv \langle a_n|_1\langle a_n|_2 \text{ tr}_F\Big [ |G_a(t)\rangle \langle G_a(t)| \Big ] |a_n\rangle _1|a_n\rangle _2$ , where $\text{ tr}_F[\bullet ]$ is the partial trace over the field degrees of freedom. Similarly, the dormant probability is defined as $p_{b_1,b_2 \rightarrow b_1,b_2}(t) \equiv \langle b_n|_1\langle b_n|_2 \text{ tr}_F\Big [ |G_b(t)\rangle \langle G_b(t)| \Big ] |b_n\rangle _1|b_n\rangle _2$. With Eq. (17), we find that $p_{a_1,b_2 \rightarrow a_1,a_2}(t) = \sum _\omega |R_{a\omega }(t)|^2$. With Eq. (18), we also find that $p_{b_1,b_2 \rightarrow b_1,b_2}(t) = \sum _\omega |D_\omega (t)|^2$.

To obtain explicit expressions, we turn to the one-dimensional real-space representation of the frequency-dependent amplitudes, namely, $R_a(z,t) \equiv \sum _\omega R_{a \omega }(t) \exp (ik_\omega z)$ and $D(z,t) \equiv \sum _\omega D_\omega (t) \exp (ik_\omega z)$, as done in Eq. (13), and find that

19 $$\begin{aligned} p_{a_1,b_2 \rightarrow a_1,a_2}(t) = \frac{1}{2\pi \varrho c}\int _{-\infty }^{\infty } |R_a(z,t)|^2 dz, \end{aligned}$$

and

20 $$\begin{aligned} p_{b_1,b_2 \rightarrow b_1,b_2}(t) = \frac{1}{2\pi \varrho c}\int _{-\infty }^{\infty } |D(z,t)|^2 dz. \end{aligned}$$

See the expressions for $R_a(z,t)$ and *D*(*z*, *t*) in the methods.

We now assume the spontaneously emitted photon as described by Eq. (15). If the initial state of the original base is $|a_n\rangle _1$, we make $\phi (z,0) = F(z,0) = \sum _{\omega } F_{\omega }(0)\exp (ik_{\omega } z)$; otherwise (for $|b_n\rangle _1$), we make $\phi (z,0) = D(z,0)$ (defined in the previous paragraph). We find that (see Methods)

21 $$\begin{aligned} p_{a_1,b_2 \rightarrow a_1,a_2}(t\rightarrow \infty ) = \mathcal {P}(\Delta , \delta _{L-bJ}), \end{aligned}$$

where the detuning is $\delta _{L-bJ} \equiv \omega _L - \omega _{bJ}$, with reference to the perturbed frequency transition $\omega _{bJ} \equiv \omega _b^{(0)} + J$, and

22 $$\begin{aligned} \mathcal {P}(\Delta , \delta _{L-bJ}) \equiv \frac{\Gamma ^2}{\left( \frac{2\Gamma -\Delta }{2}\right) ^2+\delta ^2_{L-bJ}} \left[ 1 + \frac{\Delta }{2\Gamma } - \frac{\Delta (2\Gamma + \Delta )}{\left( \frac{2\Gamma +\Delta }{2}\right) ^2+\delta ^2_{L-bJ}}\right] . \end{aligned}$$

We regard only $\Delta$ and *J* as the truly free parameters in Eq. (22). We assume, by contrast, that $\omega _L$ is fixed by the external world, whereas $\omega _b^{(0)}$ and $\Gamma$ are fixed by the internal world. We are particularly interested in the regimes where $\omega _L - \omega _b^{(0)} \gg \Gamma$ (blue-detuning) and $J>0$, which clarify the picture.

### Optimal replication

**Figure 2:**
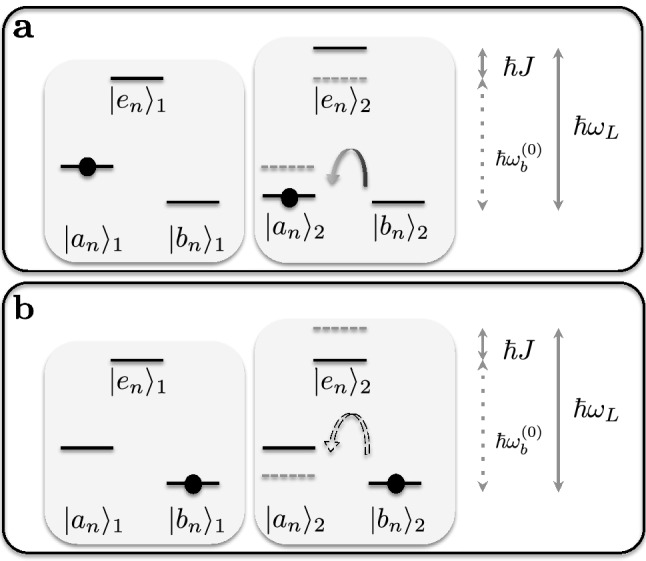
Mechanism behind replication and mutation. (**a**) A gene base at $|a_n\rangle _1$ (black dot) induces an energy shift (from the gray to the black horizontal bars) of $\hbar J$ (smaller arrow), building a resonance with the photon, $\hbar \omega _b^{(0)}+\hbar J = \hbar \omega _L$ (longer arrow). $\hbar \omega _b^{(0)}$ is the transition energy of the unperturbed lambda system (dotted arrow). The environment base thus undergoes a replication transition $|b_n\rangle _2 \rightarrow |a_n\rangle _2$ (full curved arrow). (**b**) A gene base at $|b_n\rangle _1$ (black dot) leaves the frequencies unaltered (black horizontal bars), keeping the environment base far from resonance, $\omega _b^{(0)} \ll \omega _L$. However, rare off-resonant photon absorption may induce the mutation transition $|b_n\rangle _2 \rightarrow |a_n\rangle _2$ (dashed curved arrow).

The replication probability at long times, $p_{a_1,b_2 \rightarrow a_1,a_2}(\infty )$, is maximized when the coupling $J=J(r_n)$ induces the resonance condition $\delta _{L-bJ} \equiv \omega _L - \omega _{bJ} = \omega _L - (\omega _b^{(0)} + J) = 0$. This energy-matching mechanism increasing the likelihood of the process, conditional to the distance $r_n$ between the gene base and the environment base, is reminiscent of an enzyme effect (see Fig. 2a). Perfect replication also requires a monochromatic photon ($\Delta \ll \Gamma$), as we find from Eq. (22), $p_{a_1,b_2 \rightarrow a_1,a_2}(\infty )\Big |_{\left\{ \delta _{L-bJ}=0,\ \Delta \rightarrow 0 \right\} } = \mathcal {P}(0,0) = 1$. The dormant transition probability is given by

23 $$\begin{aligned} p_{b_1,b_2 \rightarrow b_1,b_2}(\infty ) = 1-\mathcal {P}(\Delta , \delta _{L-b}), \end{aligned}$$

where the detuning now refers to the unperturbed frequency, $\delta _{L-b} \equiv \omega _L - \omega _b^{(0)}$, and $\mathcal {P}(\Delta , \delta _{L-b})$ is also defined by Eq. (22). The problem of maximizing both the replication and the dormant transition probabilities is discussed in the following.

### Mutations

Optimizing the replication transition, as described by Eq. (21), and the dormant transition, Eq. (23), requires a coupling *J* that makes the environment base resonant with the photon when the original lambda system is at $|a_n\rangle _1$ (i.e., $\omega _{bJ} = \omega _L$), whereas keeping it far from resonance when the original lambda system is at $|b_n\rangle _1$. The crucial point here is to notice that, for any finite *J*, an off-resonant photon with respect to the unperturbed frequency ($\delta _{L-b} = J$, since $\omega _{bJ} = \omega _L$) can eventually be absorbed, with the mutation probability (see Fig. 2b) $p_{b_1,b_2 \rightarrow b_1,a_2}(\infty ) = \sum _\omega |M_{a\omega }(\infty )|^2 = \mathcal {P}(\Delta , \delta _{L-b})$, which vanishes only in the $\delta _{L-b} = J \rightarrow \infty$ limit. This requires un unphysical photon of infinite frequency to fulfill the perfect replication condition, $\omega _L = \omega _{b}^{(0)}+ J$. Put differently, equations $p_{a_1,b_2 \rightarrow a_1,a_2}(\infty ) = 1$ and $p_{b_1,b_2 \rightarrow b_1, b_2}(\infty ) = 1$ cannot be simultaneously satisfied for finite couplings, $J < \infty$.

The finite off-resonant photon absorption probability, leading to $p_{b_1,b_2 \rightarrow b_1, b_2}(\infty ) < 1$, is at the root of the unexpected mutations in this model. It is unexpected because the model was not intentionally designed to present mutations. On the contrary, we intended to find perfect cloning on a preferred basis, as allowed by the no-cloning theorem^32^. Nevertheless, the idea of a light-induced rare mutation represents here an appealing analogy with natural mutations in biological genes. According to ref.^5^, error in replicating information creates the conditions for the evolvability of life as we know it. This turns an unfortunate drawback into a powerful resource.

Finally, we note that in the $r_n \rightarrow \infty$ limit (i.e., $J(\infty )=0$), the incoming photon is off-resonant for both initial states of the gene base, $\delta _{L-bJ} = \delta _{L-b} \gg \Gamma$, leading to a predominantly dormant dynamics, $p_{b_1,b_2 \rightarrow b_1,b_2}(\infty ) \rightarrow 1$ and $p_{a_1,b_2 \rightarrow a_1,a_2}(\infty ) \rightarrow 0$, as expected from the $U(r_n\rightarrow \infty ) \rightarrow 1$ condition discussed after Eq. (5).

### Dissipative adaptation

We finally investigate how dissipative adaptation underlies the self-replication in the present model. As stated in the introduction, dissipative adaptation is a general thermodynamic mechanism explaining lifelike self-organization in classical far-from-equilibrium systems. It clarifies how fine-tuned, exceptional behaviours can be fundamentally related with work consumption. The main picture is that, when a given fluctuating physical system absorbs work from its environment, it can reach exceptional dynamical transformations that are selected by the work source characteristics. If the excess energy provided by this nonequilibrium work source is dissipated to the environment (in the form of heat), the system can get irreversibly trapped in those rare configurations; in other words, adapted to its environment. Mathematically, this idea is best described in terms of a fluctuation theorem. By using Crooks’ microscopic reversibility condition for the forward, $p_{i\rightarrow j}(t)$, and backward, $p^*_{j\rightarrow i}(\tau -t)$, classical trajectory probabilities between the initial *i* and the final *j* states^43^, the dissipative adaptation has been formulated as^13,21^

24 $$\begin{aligned} \frac{p_{i\rightarrow j}(t) }{p_{i\rightarrow k}(t) } = e^{-\beta E_{kj}} \frac{p^*_{j\rightarrow i}(\tau -t) }{p^*_{k\rightarrow i}(\tau -t) } \frac{ \langle e^{-\beta W_{\mathrm {abs}} } \rangle _{ik}}{\langle e^{-\beta W_{\mathrm {abs}} } \rangle _{ij} }, \end{aligned}$$

where the angle brackets denote a weighted average over all microtrajectories with fixed start *i* and end *j*, *k* points. $\beta$ is the inverse temperature, $E_{kj} = E_j - E_k$ is the energy difference, and $W_{\mathrm {abs}}$ is the stochastic nonequilibrium work absorbed from a time-dependent drive. Equation (24) evidences that a higher work absorption in the *i* to *j* transition boosts the probability of state *j* over the alternative *k*. Dissipative adaptation can, therefore, describe the self-organization of a physical system enabling it to become apparently well suited to perform some finely-tuned, exceptional task: to seek energy^16,23^, to avoid energy^23,24,44^, or to self-replicate^12,13,21^.

Here, we find quite similar behaviour. An exceptionally high probability of replication at a zero-temperature environment requires the absorption of a proportionally high amount of average work, as given by

25 $$\begin{aligned} p_{a_1,b_2 \rightarrow a_1,a_2}(\infty ) = \langle W_{\mathrm {abs}} \rangle _{a_1,b_2}/(2\hbar \omega _L). \end{aligned}$$

$\langle W_{\mathrm {abs}} \rangle _{a_1,b_2}$ is more precisely defined in the following section; its index denotes that the average is calculated with the initial state $|a_n\rangle _1|b_n\rangle _2$ for the matter (and $|1^b\rangle$ for the field, as usual). Because Eq. (25) is valid at zero temperature and has been obtained from a fully-quantized model, we can call it a quantum dissipative adaptation^24^. We emphasize that Eq. (25) is independent of the pulse envelope function (as defined in Eq. (14)), and that it generalizes the result from ref.^24^, in being not limited to a resonant photon, and in having been obtained for the dynamics of coupled lambda systems.

Before going through the details concerning the derivation of Eq. (25), it is worth calling attention to some of its most meaningful consequences.

First, it turns out that the absorbed work is not directly proportional to the excited-state population, namely $|R_e(t)|^2$, but instead to the time integral of $|R_e(t)|^2$ [as we can see from Eq. (41)]. As a surprising consequence, while the monochromatic regime ($\Delta \rightarrow 0$) maximizes the work absorption (along with $p_{a_1,b_2 \rightarrow a_1,a_2}(\infty )$), it nonetheless minimizes the occupation of the state $|e_n\rangle _2$ [i.e., $|R_e(t)|^2 \propto \Delta \rightarrow 0$, as it can be obtained from Eq. (42)]. Such a virtual occupation of the excited state, as necessary for maximal work absorption, is a quantum-coherent process that goes beyond any classical scenario possibly described by Eq. (24). Classically, if a particle jumps from one metastable state to another one (as, for instance, in a double-well potential), there is a $100\%$ chance that it will be transiently found at the top of the hill at some instant of time.

Second, the absorbed work does not depend on the final energy stored in the system. To see this, we can substitute Eq. (22) back in Eq. (25), and notice that Eq. (22) does not depend at all on $\delta _a^{(0)}$ (as defined in Eqs.(8) and (9)). Just to provide some examples, the expression for the work simplifies, at resonance, to $\langle W_{\mathrm {abs}} \rangle _{a_1,b_2} = 2\hbar \omega _L /\left[ 1+ \Delta /(2\Gamma ) \right]$, and, in the monochromatic regime, to the Lorentzian $2\hbar \omega _L/\left[ 1 + (\delta _{L-bJ}/\Gamma )^2 \right]$. As it happens in classical thermodynamics, the work here is process-dependent and, because the lambda system is open, the work cannot be directly obtained from the variation of the internal (average) energy. When $\delta _a^{(0)} = 0$, no energy will be stored at the end, so the nonequilibrium work will have been entirely dissipated as heat to the environment.

Third, the system-plus-reservoir approach used here allowed us to express the quantum dissipative adaptation with no use of probability ratios (as they appear in the classical version, resulting from using Crooks’ condition). However, defining a stochastic work (in the sense implied by Crooks) for this single-photon scenario addressed here will be left as an open problem. This discussion will be especially relevant at finite temperatures, due to the presence of stochastic heat absorption. In the zero-temperature limit, the replication is irreversible. Once the base achieves the final state $|a_n\rangle _2$, it stays there. This final state is also transparent to any following pulses in the $\left\{ b_\omega \right\}$ modes that may arrive later. Put differently, $p_{a_1,a_2 \rightarrow a_1,b_2}(t) = 0$.

Last, but not least, casting our results in terms of a dissipative adaptation serves the main purpose of suggesting that our toy model may represent a much broader phenomenon spanning diverse classical and quantum systems, possibly comprising disordered and complex ones. The thermodynamic concept of dissipative adaptation is, perhaps, the long awaited guide for us to imitate the transition from inanimate to living matter in diverse systems, so as to understand and extend the marvellous architectures, functions, and evolution of living things^13^.

### Work consumption

To obtain our quantum version of the dissipative adaptation, Eq. (25), we depart from a definition of average incoming work as given by the Heisenberg picture, following ref.^24^,

26 $$\begin{aligned} \langle W_{\mathrm {in}} \rangle \equiv \int _0^\infty \langle \left( \partial _t d(t) \right) E_{\mathrm {in}}(t) \rangle dt, \end{aligned}$$

where $d(t) = U_n^\dagger \ d \ U_n$ is the dipole operator and $E_{\mathrm {in}}(t)$ is the incoming-field operator. Here, $d = \sum _i d_{ei} (\sigma _{i2}^\dagger + \text{ H.c.})$, $E_{\mathrm {in}}(t) = \sum _\omega i\epsilon (a_\omega + b_\omega ) e^{-i \omega t} + \text{ H.c. }$, and $d_{ei} \epsilon = \hbar g$. Definition (26) is very close to our classical notion of work^40,45^ (to see that, one can think of an initial coherent, or semiclassical, incoming pulse $|\alpha \rangle$, fulfilling $a_\omega |\alpha \rangle = \alpha _\omega |\alpha \rangle$, as established by Glauber^46^). The Heisenberg picture also provides a signature to characterize how adding the single photon pulse as described by state $|1^b\rangle$ is in general not equivalent to slightly increasing the environment temperature. Because state $|1^b\rangle$ presents coherence in the basis of frequency modes, the pure-state correlation function, namely $\langle 1^b| E_{\mathrm {in}}^{(-)}(t') E_{\mathrm {in}}^{(+)}(t) |1^b\rangle$, where $(\pm )$ is for the positive/negative frequencies^46^, is in general quite different from the equilibrium-state correlation function at temperature *T*, namely $\text{ tr }[\rho _T E_{\mathrm {in}}^{(-)}(t') E_{\mathrm {in}}^{(+)}(t)]$, where $\rho _T$ is a Gibbs state of the field with respect to $H_F$. In other words, the electromagnetic field in the pure state containing a single-photon pulse behaves more as an external driving force (a source of work) than as a stochastic Langevin force (a source of heat). In the rotating-wave approximation, and using the initial global state $|a_n\rangle _1|b_n\rangle _2|1^b\rangle$, we find that (see Methods) $\langle W_{\mathrm {in}} \rangle _{a_1,b_2} = \langle W_{\mathrm {abs}} \rangle _{a_1,b_2} + \langle W_{\mathrm {reac}} \rangle _{a_1,b_2}$, in terms of the absorptive contribution,

27 $$\begin{aligned} \langle W_{\mathrm {abs}} \rangle _{a_1,b_2} \equiv \hbar \omega _L \int _0^\infty (-2g) \mathfrak {R}[R_e^* e^{- i \delta _a^{ (0) } t} F(-ct,0)] dt, \end{aligned}$$

and the reactive (dispersive) contribution^45^,

28 $$\begin{aligned} \langle W_{\mathrm {reac}} \rangle _{a_1,b_2} \equiv \int _0^\infty (-2\hbar g) \mathfrak {R}[i R_e^* e^{- i \delta _a^{ (0) } t} (\partial _t{F}^e) e^{-i\omega _L t}] dt. \end{aligned}$$

$\mathfrak {R}[\bullet ]$ stands for the real part. In Eq. (28), we have defined $F^e(t)$ such that $F(-ct,0) \equiv F^e(t) \exp (-i\omega _L t)$. Eqs.(41) and (51) in the Methods, combined with (27), give us Eq. (25).

The meaning of the absorptive and reactive work contributions from a single-photon pulse becomes clearer in the monochromatic regime, where an analogy with a classical harmonic oscillator takes place. The monochromatic regime is set for $\Delta \ll \Gamma$, given the exponential pulse used in Eq. (21). We then find that $r(t) \approx \chi \ f(t)$, where the susceptibility is defined as $\chi \equiv i \Gamma /(\Gamma - i \delta _{L-bJ})$. Here, $r(t) \equiv \sqrt{\Gamma } R_e(t) e^{i \delta _a^{ (0) } t}$ and $f(t) \equiv i\sqrt{\Delta }\exp [-(\Delta /2 + i\omega _L)t]$. This approximately linear dependence is obtained from Eq. (42), which depends on the entire history of the photon pulse, revealing the non-Markovianity of the dynamics of the lambda systems^47^. In the monochromatic regime, however, we find that the approximate linearity holds at times $t\gg \Gamma ^{-1}$. By writing the susceptibility in terms of its real and imaginary parts, $\chi = \chi ' + i \chi ''$, we find that $\langle W_{\mathrm {abs}} \rangle _{a_1,b_2} \approx 2\hbar \omega _L \ \chi ''$, and $\langle W_{\mathrm {reac}} \rangle _{a_1,b_2} \approx \hbar \Delta \ \chi '$, in close analogy to what has been discussed in refs.^40,45^. Note that the reactive work, $\langle W_{\mathrm {reac}} \rangle _{a_1,b_2}$, vanishes both at resonance ($\delta _{L-bJ} = 0$), and arbitrarily far from resonance, $\delta _{L-bJ} \rightarrow \infty$ (as expected in dispersive light-matter interactions). More importantly, it vanishes in the monochromatic limit $\Delta \rightarrow 0$, where the self-replication is optimized. The absorptive work, $\langle W_{\mathrm {abs}} \rangle _{a_1,b_2}$, does not depend on $\Delta \rightarrow 0$, is maximal at resonance, but also vanishes far from resonance. This tells us that the far-from-resonance (rare) mutations absorb a very small (though finite) amount of work. In the context of adaptation, this means that mutations may be a source of open-endedness (that may describe evolutionary divergence and novelty) while still fulfilling a dissipative adaptation (more tightly bound to the notions of evolutionary convergence and stability).

Analogous results arise from considering a fully classical damped harmonic oscillator with complex position $r_c(t)$, driven by the force $f_c(t)=\sqrt{\Delta }\exp [-(\Delta /2 + i\omega _L) t]$, with a slowly-varying amplitude $\Delta \ll \Gamma$, where $\Gamma$ is the oscillator dissipation rate. We then have from Newtonian dynamics that $r_c(t) \approx \chi _c f_c(t)$, so the classical work reads $W_{\mathrm {cl}} \equiv \int _0^\infty 2\mathfrak {R}[f_c^* \partial _t r_c]dt = - \int _0^\infty 2\mathfrak {R}[r_c^* \partial _t f_c]dt \approx \Delta \chi _c' + 2\omega _L \chi _c''$, where $\Delta \chi _c'$ is the reactive (dispersive) part, and $2\omega _L \chi _c''$ is the absorptive part (see ref.^40^).

## Discussion

In summary, the toy model devised here shows how the nonequilibrium work provided by spontaneously emitted single-photon pulses can fuel the self-replication of an elementary quantum artificial organism formed by a chain of lambda systems. The guiding intuition was that dissipative adaptation could result in some kind of self-organized process reminding us of an artificial-life code. Quantum mechanics allowed us to think in terms of basic resources in nature, namely, atoms, photons, and their interactions.

Mutations were found to be unavoidable, though rare, due to far-from-resonance photon absorption. Interestingly, because mutations and self-replication may imply a possible route towards open-ended evolution in biological systems, the model thus alludes to a theoretical link between dissipative adaptation and open-endedness that calls for further investigations. Finally, it is worth emphasizing that the mutations play a central role in justifying a posteriori why we could assume the existence of an arbitrary state $|\mathrm {gene}\rangle _1$ from the outset. Put differently, how could state $|a_n\rangle _1$ first have appeared, in an otherwise zero-temperature autonomous universe at thermal equilibrium? Without mutations, all the bases would perpetually remain in their ground states $|b_n\rangle _i$, implying a completely dormant, rather trivial universe.

As a perspective, we can search for self-organized artificial-life codes with some degree of (quantum or classical) complexity^48^. We can think, for instance, of mimicking the self-organized evolution of an entire artificial genetic code, going beyond the artificial gene we have considered. To evolve the natural genetic code, biological organisms have taken great advantage from the so called horizontal gene transfer (HGT), according to refs.^3,49^. An artificial HGT can be envisioned by letting the free parameters $r_n$ here (representing the distances between pairs of lambda systems) to behave as Brownian particles in a common environment (to be more precise, the center of mass of each lambda system could be considered as a Brownian particle). Common environments can mediate attractive effective couplings between pairs of Brownian particles (classical and quantum), as shown in refs.^50–52^. The size *N* of each artificial gene would then become a stochastic variable (the artificial gene being slowly split or merged with others in the environment), simply from the environment-induced Brownian movements and effective couplings, thus implementing an artificial HGT.

## Methods

### Solution of the global dynamics

The Schrödinger equation $i\hbar \partial _t |G_k(t)\rangle = H|G_k(t)\rangle$ (as defined in Eqs.(7) and (16)) leads us to

29 $$\begin{aligned} \partial _t D_\omega&= -i\omega D_\omega + g M_e, \end{aligned}$$

30 $$\begin{aligned} \partial _t M_e&= -i\omega _b^{(0)} M_e - g \sum _\omega (D_\omega + M_{a\omega }), \end{aligned}$$

31 $$\begin{aligned} \partial _t M_{a\omega }&= -i(\omega + \delta _a^{(0)}) M_{a\omega } + g M_e, \end{aligned}$$

for $$k = a$$, and

32 $$\begin{aligned} \partial _t F_\omega&= -i(\omega +\delta _a^{(0)}) F_\omega + g R_e, \end{aligned}$$

33 $$\begin{aligned} \partial _t R_e&= -i(\delta _a^{(0)} + \omega _b^{(0)} + J) R_e - g \sum _\omega (F_\omega + R_{a\omega }), \end{aligned}$$

34 $$\begin{aligned} \partial _t R_{a\omega }&= -i(\omega + 2\delta _a^{(0)} - J) R_{a\omega } + g R_e, \end{aligned}$$

for $$k=b$$.

Formally integrating for $D_\omega (t)$, and using $M_{a\omega }(0) = 0$, gives, in the Wigner-Weisskopf approximation, that

35 $$\begin{aligned} \partial _t M_e = -(\Gamma +i\omega _b^{(0)}) M_e - g D(-ct,0), \end{aligned}$$

where $\Gamma \equiv 2\pi \varrho g^2$ (due to the continuum limit, $\sum _\omega \rightarrow \varrho \int d\omega$), and $D(z,t) \equiv \sum _\omega D_\omega (t)\exp (ik_\omega z)$ (where $\omega = c k_\omega$). Also, we find that $D(z,t) = D(z-ct,0) + \sqrt{2\pi \varrho \Gamma } \Theta (z) \Theta (t-z/c) M_e(t-z/c)$, and that

36 $$\begin{aligned} M_a(z,t) = \sqrt{2\pi \varrho \Gamma } \Theta (z) \Theta (t-z/c) M_e(t-z/c) e^{-i\delta _a^{(0)}z/c}. \end{aligned}$$

The results for $R_e(t)$, *F*(*z*, *t*) and $R_a(z,t)$ follow quite similarly,

37 $$\begin{aligned} \partial _t R_e = -\left( \Gamma +i(\omega _{bJ} +\delta _a^{(0)}) \right) R_e - g F(-ct,0), \end{aligned}$$

where $$\omega _{bJ} \equiv \omega _b^{(0)} + J$$, $$F(z,t) = F(z-ct,0) e^{-i\delta _a^{(0)} t} + \sqrt{2\pi \varrho \Gamma } \Theta (z) \Theta (t-z/c) R_e(t-z/c) e^{-i\delta _a^{(0)} z/c}$$, and

38 $$\begin{aligned} R_a(z,t) = \sqrt{2\pi \varrho \Gamma } \Theta (z) \Theta (t-z/c) R_e(t-z/c) e^{ -i(2\delta _a^{(0)} - J) z/c}. \end{aligned}$$

The mutation probability reads

39 $$\begin{aligned} p_{b_1,b_2 \rightarrow b_1,a_2}(t) = \sum _\omega |M_{a\omega }(t)|^2 = \frac{1}{2\pi \varrho c}\int _{-\infty }^\infty |M_a(z,t)|^2 dz. \end{aligned}$$

By substituting Eq. (36) in (39), and changing variables, we find that $p_{b_1,b_2 \rightarrow b_1,a_2}(t) = \Gamma \int _{0}^{t} |M_e(t')|^2 dt'$. Similarly, by substituting Eq. (38) in the replication probability, namely,

40 $$\begin{aligned} p_{a_1,b_2 \rightarrow a_1,a_2}(t) = \frac{1}{2\pi \varrho c}\int _{-\infty }^{\infty } |R_a(z,t)|^2 dz, \end{aligned}$$

we find that

41 $$\begin{aligned} p_{a_1,b_2 \rightarrow a_1,a_2}(t) = \Gamma \int _{0}^{t} |R_e(t')|^2 dt'. \end{aligned}$$

The integration above is performed with the solution of $R_e(t)$, that is,

42 $$\begin{aligned} R_e(t) = - g \int _0^t F(-ct',0) e^{-\left( \Gamma + i(\omega _{bJ}+\delta _a^{(0)}) \right) (t-t')} dt'. \end{aligned}$$

Eqs.(14) and (15) allow $R_e(t)$ to be analytically obtained. Eq. (41) is a key step for the quantum dissipative adaptation relation, Eq. (25), as explained below.

### Calculating the incoming work

Using integration by parts, we can rewrite Eq. (26) as $\langle W_{\mathrm {in}} \rangle = -\int _0^\infty \langle d(t) \partial _t E_{\mathrm {in}}(t) \rangle dt$. In the present model, this results in

43 $$\begin{aligned} \langle W_{\mathrm {in}}\rangle = -\int _0^\infty dt (i\hbar g) \sum _\omega (-i\omega ) \langle \sigma _{a2}^\dagger (t) a_\omega \rangle e^{-i\omega t} + (-i\omega ) \langle \sigma _{b2}^\dagger (t) b_\omega \rangle e^{-i\omega t} + \text{ c.c. }, \end{aligned}$$

where $\text{ c.c }$ stands for complex conjugate. Choosing $|1^b\rangle$ as the initial state of the field implies that $\langle \sigma _{a2}^\dagger (t) a_\omega \rangle = 0$. The non-zero correlation function gives

44 $$\begin{aligned} \langle \sigma _{b2}^\dagger (t) b_\omega \rangle&= \langle U^\dagger \sigma _{b2}^\dagger U b_\omega |a_n\rangle _1|b_n\rangle _2|1^b\rangle \end{aligned}$$

45 $$\begin{aligned}&= \langle U^\dagger \sigma _{b2}^\dagger U F_\omega (0) |a_n\rangle _1|b_n\rangle _2|0\rangle \end{aligned}$$

46 $$\begin{aligned}&= \langle U^\dagger \sigma _{b2}^\dagger e^{-i\delta _a^{(0)} t} F_\omega (0) |a_n\rangle _1|b_n\rangle _2|0\rangle \end{aligned}$$

47 $$\begin{aligned}&= \langle U^\dagger e^{-i\delta _a^{(0)} t} F_\omega (0) |a_n\rangle _1|e_n\rangle _2|0\rangle \end{aligned}$$

48 $$\begin{aligned}&= \langle G_a(t)| \left( |a_n\rangle _1|e_n\rangle _2|0\rangle \right) e^{-i\delta _a^{(0)} t} F_\omega (0) \end{aligned}$$

49 $$\begin{aligned}&= R_e^*(t) e^{-i\delta _a^{(0)} t} F_\omega (0). \end{aligned}$$

Finally,

50 $$\begin{aligned} \langle W_{\mathrm {in}}\rangle = -\hbar g \int _0^\infty R_e^*(t) e^{-i\delta _a^{(0)} t} \left( i \partial _t F(-ct,0) \right)dt + \text{ c.c. }. \end{aligned}$$

We define $F(-ct,0) = F^e(-ct,0)\exp (-i\omega _L t)$, and obtain Eqs.(27) and (28). To find Eq. (25), we get from Eq. (37) that

51 $$\begin{aligned} \partial _t |R_e(t)|^2 = -2\Gamma |R_e(t)|^2 - 2 g \mathfrak {R}[R_e^*(t) e^{-i\delta _a^{ (0) } t } F(-ct,0)]. \end{aligned}$$

We substitute Eq. (51) in Eq. (41), using that $R_e(0) = R_e(\infty ) = 0$. Finally, we identify the real part in Eq. (51) with that in the absorptive term, Eq. (27), which follows from Eq. (50). This leads to Eq. (25), independently of the choice for $F(-ct,0)$, that is, the initial photon wavepacket.

## Data Availability

Data sharing not applicable to this article as no datasets were generated or analysed during the current study.

## References

[1] Schrödinger, E. *What is Life? The Physical Aspect of the Living Cell* (Cambridge University Press, 1944).

[2] Rasmussen S (2004). Transitions from nonliving to living matter. Science.

[3] Cleland, C. E. *The Quest for a Universal Theory of Life: Searching for Life as We Don’t Know It* (Cambridge University Press, 2019).

[4] England, J. *Every Life is on Fire: How Thermodynamics Explains the Origins of Living Things* (Basic Books, 2020).

[5] Goldenfeld N, Biancalani T, Jafarpour F (2017). Universal biology and the statistical mechanics of early life. Philos. Trans. R. Soc. A.

[6] Lenski RE, Ofria C, Pennock RT, Adami C (2003). The evolutionary origin of complex features. Nature.

[7] Packard N (2019). An overview of open-ended evolution: Editorial introduction to the open-ended evolution ii special issue. Artif. Life.

[8] Alvarez-Rodriguez U, Sanz M, Lamata L, Solano E (2014). Biomimetic cloning of quantum observables. Sci. Rep..

[9] Alvarez-Rodriguez U, Sanz M, Lamata L, Solano E (2016). Artificial life in quantum technologies. Sci. Rep..

[10] Alvarez-Rodriguez U, Sanz M, Lamata L, Solano E (2018). Quantum artificial life in an IBM quantum computer. Sci. Rep..

[11] Lamata L (2020). Quantum machine learning and quantum biomimetics: A perspective. Mach. Learn. Sci. Technol..

[12] England JL (2013). Statistical physics of self-replication. J. Chem. Phys..

[13] England J (2015). Dissipative adaptation in driven self-assembly. Nat. Nanotechnol..

[14] Carnall JMA (2010). Mechanosensitive self-replication driven by self-organization. Science.

[15] Ito S (2013). Selective optical assembly of highly uniform nanoparticles by doughnut-shaped beams. Sci. Rep..

[16] Kondepudi D, Kay B, Dixon J (2015). End-directed evolution and the emergence of energy-seeking behaviour in a complex system. Phys. Rev. E.

[17] Bachelard N (2017). Emergence of an enslaved phononic bandgap in a non-equilibrium pseudo-crystal. Nat. Mater..

[18] Ropp C, Bachelard N, Barth D, Wang Y, Zhang X (2018). Dissipative self-organization in optical space. Nat. Photon..

[19] Sarkar S, England JL (2019). Design of conditions for self-replication. Phys. Rev. E.

[20] te Brinke E (2020). Dissipative adaptation in driven self-assembly leading to self-dividing fibrils. Nat. Nanotechnol..

[21] Perunov N, Marsland RA, England JL (2016). Statistical physics of adaptation. Phys. Rev. X.

[22] Horowitz JM, England JL (2017). Spontaneous fine-tuning to environment in many-species chemical reaction networks. Proc. Natl. Acad. Sci. USA.

[23] Kachman T, Owen JA, England JL (2017). Self-organized resonance during search of a diverse chemical space. Phys. Rev. Lett..

[24] Valente D, Brito F, Werlang T (2021). Quantum dissipative adaptation. Comm. Phys..

[25] Sadownik JW, Mattia E, Nowak P, Otto S (2016). Diversification of self-replicating molecules. Nat. Chem..

[26] Adams A, Zenil H, Davies PCW, Walker SI (2017). Formal definitions of unbounded evolution and innovation reveal universal mechanisms for open-ended evolution in dynamical systems. Sci. Rep..

[27] Andrieux D, Gaspard P (2008). Nonequilibrium generation of information in copolymerization processes. Proc. Natl. Acad. Sci. USA.

[28] Jarzynski C (2008). The thermodynamics of writing a random polymer. Proc. Natl. Acad. Sci. USA.

[29] Parrondo JMR, Horowitz JM, Sagawa T (2015). Thermodynamics of information. Nat. Phys..

[30] Walker IS, Davies PCW (2013). The algorithmic origins of life. J. R. Soc. Interface.

[31] Conte, T. *et al.**Thermodynamic computing*. arXiv:1911.01968 (2019).

[32] Wootters WK, Zurek WH (1982). A single quantum cannot be cloned. Nature.

[33] Buzek V, Hillery M (1996). Quantum copying: Beyond the no-cloning theorem. Phys. Rev. A.

[34] Gisin N, Massar S (1997). Optimal quantum cloning machines. Phys. Rev. Lett..

[35] Valente D (2012). Universal optimal broadband photon cloning and entanglement creation in one-dimensional atoms. Phys. Rev. A.

[36] Bryngelson JD, Wolynes PG (1987). Spin glasses and the statistical mechanics of protein folding. Proc. Natl. Acad. Sci. USA.

[37] Valente D, Werlang T (2020). Frustration and inhomogeneous environments in relaxation of open chains with Ising-type interactions. Phys. Rev. E.

[38] Guerreiro T (2021). Quantum molecular robots. Quantum. Sci. Technol..

[39] Guerreiro, T. *Molecular machines for quantum error correction*. arXiv:2103.05184 (2021).

[40] Cohen-Tannoudji, C., Dupont-Roc, J. & Grynberg, G. *Atom–Photon Interactions* (Wiley, 2004).

[41] Oviedo-Casado S (2016). Phase-dependent exciton transport and energy harvesting from thermal environments. Phys. Rev. A.

[42] Tomasi S, Baghbanzadeh S, Rahimi-Keshari S, Kassal I (2019). Coherent and controllable enhancement of light-harvesting efficiency. Phys. Rev. A.

[43] Crooks GE (1999). Entropy production fluctuation theorem and the nonequilibrium work relation for free energy differences. Phys. Rev. E.

[44] Kedia, H., Pan, D., Slotine, J. J. & England, J. L. *Drive-specific adaptation in disordered mechanical networks of bistable springs*. arXiv:1908.09332 (2019).

[45] Valente D, Brito F, Ferreira R, Werlang T (2018). Work on a quantum dipole by a single-photon pulse. Opt. Lett..

[46] Glauber RJ (1963). Coherent and incoherent states of the radiation field. Phys. Rev..

[47] Valente D, Arruda MFZ, Werlang T (2016). Non-Markovianity induced by a single-photon wave packet in a one-dimensional waveguide. Opt. Lett..

[48] Hillberry, L. E. *et al.**Entangled quantum cellular automata, physical complexity, and goldilocks rules*. arXiv:2005.01763 (2020).

[49] Vetsigian K, Woese C, Goldenfeld N (2006). Collective evolution and the genetic code. Proc. Natl. Acad. Sci. USA.

[50] Duarte OS, Caldeira AO (2006). Effective coupling between two Brownian particles. Phys. Rev. Lett..

[51] Duarte OS, Caldeira AO (2009). Effective quantum dynamics of two Brownian particles. Phys. Rev. A.

[52] Valente DM, Caldeira AO (2010). Thermal equilibrium of two quantum Brownian particles. Phys. Rev. A.

